# Insecticidal effects of deltamethrin in laboratory and field populations of *Culicoides* species: how effective are host-contact reduction methods in India?

**DOI:** 10.1186/s13071-017-1992-0

**Published:** 2017-01-31

**Authors:** Rien De Keyser, Clare Cassidy, Swathi Laban, Prakash Gopal, John A. Pickett, Yarabolu K. Reddy, Minakshi Prasad, Gaya Prasad, Sreekumar Chirukandoth, Kandasamy Senthilven, Simon Carpenter, James G. Logan

**Affiliations:** 1 Department of Disease Control, Faculty of Infectious and Tropical Diseases, London School of Hygiene and Tropical Medicine, Keppel Street, London, WC1E 7HT UK; 2 Vaccine Research Centre-Viral Vaccines, Centre for Animal Health Studies, Tamil Nadu Veterinary and Animal Sciences University, Madhavaram Milk Colony, Chennai, 600 051 India; 3 Rothamsted Research, West Common, Harpenden, Hertfordshire AL5 2JQ UK; 4 Department of Animal Biotechnology, College of Veterinary Science, Lala Lajpat Rai University of Veterinary and Animal Sciences, Hisar, 125004 Haryana India; 5 Indian Council of Agricultural Research, New Delhi, 110 001 India; 6 Postgraduate Research Institute in Animal Sciences, Kattupakkam, 603 203 India; 7 Veterinary University Training and Research Centre, Karur, 639 006 India; 8 Vector-borne viral diseases Programme, The Pirbright Institute, Ash Road, Woking, GU24 0NF UK

**Keywords:** Bluetongue virus, Insecticide, Ceratopogonidae, BTV, Ectoparasite, Vector-borne disease, Arbovirus

## Abstract

**Background:**

Bluetongue virus (BTV) is transmitted by *Culicoides* biting midges and causes bluetongue (BT), a clinical disease observed primarily in sheep. BT has a detrimental effect on subsistence farmers in India, where hyperendemic outbreaks impact on smallholdings in the southern states of the country. In this study, we establish a reliable method for testing the toxic effects of deltamethrin on *Culicoides* and then compare deltamethrin with traditional control methods used by farmers in India.

**Results:**

Effects of deltamethrin were initially tested using a colonised strain of *Culicoides nubeculosus* Meigen and a modified World Health Organisation exposure assay. This method was then applied to field populations of *Culicoides* spp. in India. The field population of *C. oxystoma* in India had a greater LC_50_ (0.012 ± 0.009%) for deltamethrin than laboratory-reared *C.nubeculosus* (0.0013 ± 0.0002%). Exposure of *C. nubeculosus* to deltamethrin at higher ambient temperatures resulted in greater rates of knockdown but a lower mortality rate at 24 h post-exposure. Behavioural assays with *C. nubeculosus* in WHO tubes provided evidence for contact irritancy and spatial repellence caused by deltamethrin. The field experiments in India, however, provided no evidence for repellent or toxic effects of deltamethrin. Traditional methods such as the application of neem oil and burning of neem leaves also provided no protection.

**Conclusions:**

Our study demonstrates that field-collected *Culicoides* in India are less susceptible to deltamethrin exposure than laboratory-bred *C. nubeculosus* and traditional methods of insect control do not provide protection to sheep. These low levels of susceptibility to deltamethrin have not been recorded before in field populations of *Culicoides* and suggest resistance to synthetic pyrethrioids. Alternative insect control methods, in addition to vaccination, may be needed to protect Indian livestock from BTV transmission.

**Electronic supplementary material:**

The online version of this article (doi:10.1186/s13071-017-1992-0) contains supplementary material, which is available to authorized users.

## Background

Bluetongue virus (BTV) is an arbovirus of international veterinary importance and is the aetiological agent of bluetongue (BT), a disease of ruminants classified as notifiable by the World Organisation for Animal Health (OIE). BTV is transmitted biologically between ruminants and certain species of deer by *Culicoides* biting midges (Diptera: Ceratopogonidae) [1, 2]. Infection with BTV can result in morbidity and mortality, particularly in sheep, but cattle and goats can also exhibit clinical signs during disease outbreaks. In India, hyperendemic outbreaks of BTV have a significant impact on subsistence sheep farmers in a region where 12% of meat production relies on sheep and goat farms [3]. BTV is endemic across the southern states of India and a total of 21 co-circulating serotypes have been identified in the country, including several exotic strains that are thought to have been introduced during sheep improvement initiatives [4].

While safe and efficacious single-serotype inactivated vaccines for BTV are now available, limited cross-protection between serotypes and their cost inhibit use on a large scale, although the recent development of an inactivated pentavalent vaccine may eventually increase uptake in poorer communities [5]. Where these vaccines are either unavailable or unaffordable, *Culicoides* control and animal movement restrictions are the only available responses to BT outbreaks [6, 7]. Control includes insecticidal applications to sheep or the use of traditional methods such as burning of plant material to reduce biting rates. No quantitative data currently exist to assess the probable impact of such measures despite their widespread use and selection of appropriate measures is largely based on anecdotal experience and cost.

Worldwide, the active ingredients used most frequently in insecticidal formulations applied to sheep are synthetic pyrethroids and these have received most attention to date in efficacy testing against *Culicoides*. In the most complete set of trials, conducted in the USA, permethrin was shown to be highly effective in laboratory and semi-field trials in killing the primary BTV vector, *Culicoides sonorensis* Wirth & Jones, but subsequently failed to prevent transmission of BTV when applied in an endemic field scenario [8, 9]. The northern European outbreak of BTV in 2006 has also led to laboratory and field testing of *Culicoides* vector control methods, primarily deltamethrin in different formulations (see [6] for review). Exposure of *Culicoides* to fleece taken from treated animals, or through direct feeding on sheep led to significant levels of mortality when compared to unexposed controls [10, 11], but this effect is often transient. Additionally, these studies are difficult to interpret because they used different testing protocols with varying exposure times to insecticides and different temperatures. Since variation in temperature and exposure time can affect efficacy of insecticides [12], these parameters need to be better understood and controlled.

In contrast to synthetic pyrethroids, Indian farmers also apply ethno-veterinary practices, including products derived from the neem tree, *Azadirachta indica* [13]. Azadirachtin, the active ingredient of neem tree leaves and seed kernels, has repellent properties and affects insect growth, fertility and oviposition [14]. However, quantitative evidence for the effectiveness of neem in controlling *Culicoides* is not currently available.

The aim of this study was initially to examine the impact of exposure time and temperature on insecticide efficacy using laboratory-reared *Culicoides nubeculosus* in the UK. From this, a standard method was developed and used to test traditional control methods in the field in India.

## Methods

### Study species

A laboratory strain of *C. nubeculosus* was used during all trials. The midges were reared at the Pirbright Institute (UK) and sent as pupae to the London School of Hygiene and Tropical Medicine for testing. Insects were maintained in an insectary at 21–22 °C with a sugar source of cotton wool soaked in 10% sucrose solution until testing. Test subjects were 1–3 day-old, females that had not been previously blood-fed.

Experiments with field populations of *Culicoides* were carried out at the Postgraduate Research Institute in Animal Sciences in Kattupakkam in India (Lat/Long: 12.83, 80.03) in June 2012 and in February 2014. Live *Culicoides* were collected using an Onderstepoort Veterinary Institute design light-suction trap with a UV tube and a damp cloth and tissue paper in the collection pot. Insects were collected between 18:00 and 8:30 h and on the morning following collection *Culicoides* were isolated, transferred to a holding container and given access to 10% sucrose solution.

### Preparation of equipment

A modified version of the World Health Organisation Pesticide Evaluation Scheme (WHOPES) general protocol for insecticide testing [15] was performed using standard WHO tubes with finer muslin material replacing the original mesh screen caps. The methods that were used in this investigation followed the protocol of Venail et al. [16]. Technical grade deltamethrin (Sigma-Aldrich, London, UK) was mixed with an acetone-silicon solvent (67% acetone, 33% silicon) to produce serial dilutions (see below). Each deltamethrin solution (2 ml) was pipetted evenly onto a Whatman n°1 filter paper (12 × 15 cm) as described in Venail et al. [16]. Control papers were impregnated with the acetone-silicon solvent mixture (2 ml). Treated papers were left to dry overnight and used within 48 h (UK experiments) or wrapped in tin foil and labelled and stored in the fridge (± 4 °C), where they were kept until transfer to India.

#### Experiments with laboratory-reared C. nubeculosus (UK experiments)

##### Dose response with WHO tubes

Approximately 20 *C. nubeculosus* were transferred into the green end of each WHO tube. Papers with different concentrations of deltamethrin (0.05%, 0.025%, 0.01%, 0.005%, 0.0025%, 0.001%, 0.0005%, 0.0001%), or a control paper, were inserted into the red end of each tube. Midges were then exposed to insecticide-treated papers for 1 h with the tubes placed in a vertical position, with the red end facing down. Knockdown was recorded after 1 h and mortality was recorded 24 h post-exposure. Knockdown was defined as midges being unable to remain standing when the tube was gently tapped. Three replicates were carried out at each concentration of deltamethrin.

##### Effect of exposure period and temperature

Two concentrations of deltamethrin-treated papers were used during these trials: one which caused a high rate of mortality in *C. nubeculosus* (0.005%) and one which caused a lower rate (0.001%). Twenty female *C. nubeculosus* were transferred to a WHO tube containing either a deltamethrin-treated or untreated control paper, using the methods described above. Two temperatures (13 °C and 25 °C) were tested with an exposure period of 20 min; three exposure periods (10 min, 20 min and 60 min) were tested and knockdown immediately after exposure and 24-h mortality was recorded. The higher temperature is representative of typical evening temperatures in South India; the lower of UK summer night time temperatures. The shorter exposure periods test for efficacy of deltamethrin under more realistic scenarios (*Culicoides* spend 10–20 min on a host [17]).

##### Contact irritancy

**Fig. 1:**
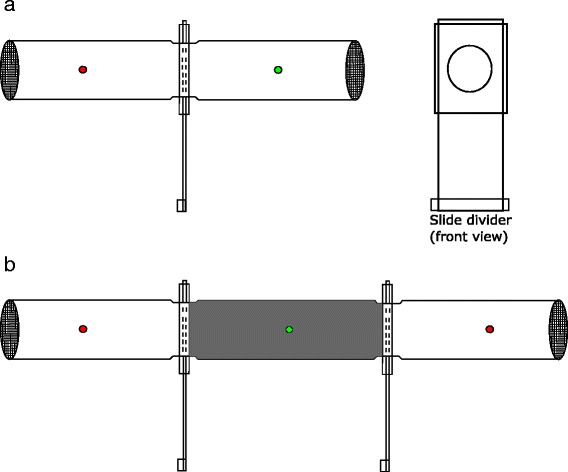
Design for testing of contact irritancy (**a**) and spatial repellency (**b**) of *Culicoides* midges to deltamethrin. To test contact irritancy, an insecticide treated paper was inserted in one end of the tube (*red* dot); a control paper was inserted in the other end (*green* dot). 20 *Culicoides* midges were released in the red end with the slider between the two tubes open (**a**). After 10 min, the number of *Culicoides* in each part of the tube was recorded. For the spatial repellency test, insects are released in a darkened centre tube (*green* dot); an insecticide treated paper or blank paper was inserted in the end tubes (*red* dots) (**b**). After 10 min, the number of *Culicoides* in each part of the tube was recorded

Contact irritancy (movement away from treated papers after contact) of *C. nubeculosus* to deltamethrin was investigated using WHO tubes with an open slide divider following the protocol of Grieco et al. [18] (Fig. 1a). This allowed *Culicoides* to move between the insecticide-treated and untreated end of the tube. Twenty *C. nubeculosus* were transferred to the red end of a WHO tube (containing deltamethrin-treated paper), which was placed horizontally in a dark box with openings at either end to allow light to enter at both ends of the tube and stimulate movement. After 10 min the slide divider was moved to the closed position and the number of *C. nubeculosus* in each chamber was recorded. Those in the clear chamber were classed as “escaping” while those in the treatment area were classed as “remaining”. The orientation of the tube was alternated in order to avoid positional bias between each experiment.

##### Spatial “repellency”

We used an assay similar to the high-throughput screening system (HITSS) created by Grieco et al. [18] to measure spatial repellency. Three WHO tubes (two red-labelled tubes and one darkened green-labelled tube) were connected with two slide dividers (Fig. 1b). An insecticide-treated paper was inserted in the end of one of the red-labelled tubes and a control paper was inserted into the other red-labelled tube. The middle green-labelled tube was covered in a black plastic material to block out light. A light source was placed at both ends of the assay to stimulate movement away from the green centre tube towards either the insecticide-treated paper or the control paper. Approximately 20 *C. nubeculosus* were transferred to the green-labelled chamber and allowed to settle for 30 s before the slide doors to the red tubes were simultaneously opened. After 10 min the slide doors were closed and the number of *C. nubeculosus* (including those knocked down) in each chamber was recorded.

#### Experiments with field-caught *Culicoides* in Tamil Nadu, India

##### Dose response

In the field in India, the following deltamethrin solutions were tested: 0.1%, 0.05%, 0.01%, 0.005%, 0.001%, 0.0005%, 0.0001%. During the 1 h exposure, the tubes were placed in a horizontal position as described in Venail et al. [17]. Approximately 25 live, field-caught *Culicoides* were transferred directly from the holding container to the green end of each WHO tube and were identified to species level following the experiment. After exposure, knockdown was recorded and insects were transferred into paper cups with access to 10% sucrose solution on cotton wool. Mortality was recorded at 6 h and 24 h post-exposure. Temperature and humidity were recorded using a data logger (TR-73U, T and D).

##### Repellent effect following exposure to different treatments in the field

The effect of traditional vector control methods used by farmers in southern India was investigated in two separate experiments. In the first experiment, 10 Madras Red sheep, a local breed (5 males, 5 females), were treated with formulations of either deltamethrin or neem oil. In the second experiment, 10 sheep (5 males, 5 females) were treated with neem oil or were placed in a pen with neem smoke released nearby. Groups of sheep for each treatment were kept in separate pens. In each experiment 10 Madras Red sheep (5 males, 5 females) were left untreated as a control in a separate pen. All animals were vaccinated against bluetongue virus using a pentavalent vaccine (produced at TANUVAS), were regularly dewormed and had not been treated with any insecticide 3 months prior to the experiment.

For application of the treatments, deltamethrin (Butox 12.5) was diluted to 2 ml/l following the manufacturer’s instructions and sheep were dipped in a tank containing a 0.2% solution. The deltamethrin treatment was applied once only. Undiluted neem oil (S.R. Neem Agro Products, Thiruchengode, India) was applied to the lateral sides of each sheep (1.5 ml on each side) and rubbed into the fleece. This process was repeated weekly during the experiment to maintain activity following manufacturer’s guidance. Neem smoke was produced by placing terracotta pots filled with neem leaves (approximately 1 kg weight of which 70% of leaves were fully dried, 20% were partially dried and 10% were freshly picked) on a fire of wood and dried leaves. Fully dried leaves were obtained by placing the leaves in direct sunlight for at least 1 day; partially dried leaves were left to dry for 2 h only prior to burning. Two pots were placed on opposite sides of each pen and smoke was produced for 2–3 h each night, starting at 18:30 h (dusk), following traditional methods.

In each pen (7.3 × 9 m) a UV CDC light-suction trap model 912 (John Hock, Gainesville, USA) was installed (1.5 m height) and *Culicoides* were collected between 18:00 and 8:00 h every night. The distance between the pens was at least 40 m in all cases to minimise interference between treatments. Each night, treatments were randomised between the different pens, using a Latin square design. *Culicoides* collected in the light-suction traps were transferred to 70% ethanol for sorting, identification and counting. Temperature and wind direction were also recorded.

##### Effect of animal treatments on Culicoides survival

Hair (1 g) was clipped from a randomly chosen sheep of each deltamethrin-treated group each day and placed in 150 ml paper cups covered with fine mesh. Approximately 30 live, wild-caught, *Culicoides* were transferred to the cups and exposed to the hair for 3 min. The cups were placed in a dark box to avoid insects staying at the top of the cup due to phototaxis and encourage contact with the hair. Following exposure, *Culicoides* knockdown was recorded and insects were transferred to clean paper cups, placed in the lab with access to 10% sucrose solution on cotton wool. Mortality was recorded 6 and 10 h post-exposure and the midges were identified to species level.

### Analyses

Only replicates with less than 20% mortality in the controls were included in the analyses. Knockdown was recorded directly after exposure and 1 h later for all tests, but was only included in results where significant effects were found. The proportions of live and dead *Culicoides* were analysed using a generalised log-logistic model with a binomial distribution, following the protocol of Ritz et al. [19]. In the exposure time and temperature experiments with *C. nubeculosus* and in the sheep hair experiment with field-collected Indian *Culicoides*, untransformed data were modelled using generalised linear models (GLM) with a binomial distribution. Chi-square tests were used for the analysis of the contact irritancy assay and spatial repellency assay to compare the number of *C. nubeculosus* responding to the treatment compared to the number responding in the control experiment. The effect of control methods in India (neem oil, neem smoke, deltamethrin) was investigated using a restricted maximum likelihood (REML) approach for both the total number of *Culicoides* and female *Culicoides*. Pen location and location nested in week were used as random design terms. Treatment and Day, and their interaction, were tested as fixed effects. All analyses were carried out using R v.3.03 [20].

## Results

### Experiments with laboratory reared *C. nubeculosus* (UK experiments)

#### Dose response

**Fig. 2:**
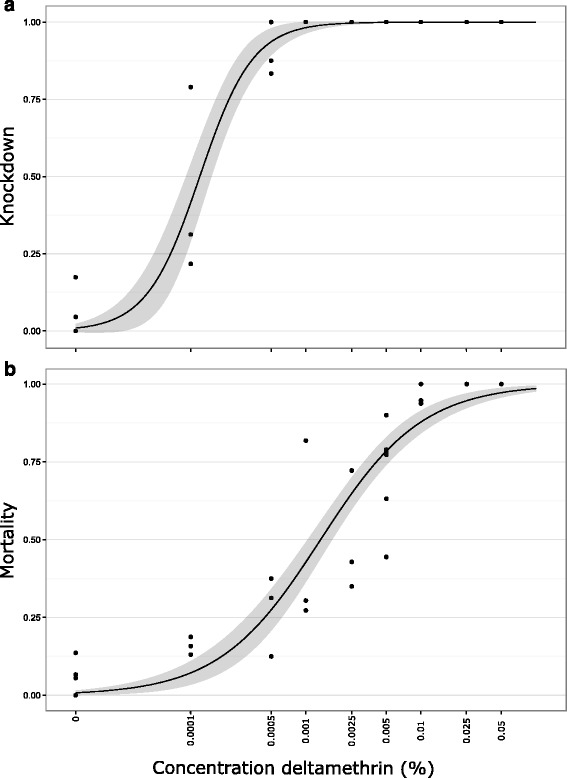
Knockdown and mortality of laboratory reared *Culicoides nubeculosus*. Knockdown of laboratory reared *C. nubeculosus* recorded 1 h after exposure to deltamethrin papers in WHO tubes (**a**) and mortality recorded 24 h after exposure (**b**). Knockdown was assessed as an insect not able to remain on its feet after gently tapping the tube. Grey bands indicate 95% confidence intervals

Deltamethrin had a dose-dependent effect on the knockdown and mortality of *Culicoides* (Fig. 2a, b). A high rate of knockdown was found after 1 h exposure, even when using low concentrations of deltamethrin, producing a LC_50_ of 1.20*10^-4^% [8.94*10^-5^, 2.00*10^-4^] (0.043 mg/m^2^) and an LC_90_ of 3.85*10^-4^% [2.56*10^-4^, 5.00*10^-4^] (0.139 mg/m^2^). The LC_50_ and LC_90_ for mortality were greater (LC_50_: 1.34*10^-3^% [1.02*10^-3^, 1.70*10^-3^] (0.468 mg/m^2^); LC_90_:1.25*10^-2^% [7.83*10^-3^, 1.72*10^-2^] (4.68 mg/m^2^).

#### Effect of exposure time

**Fig. 3:**
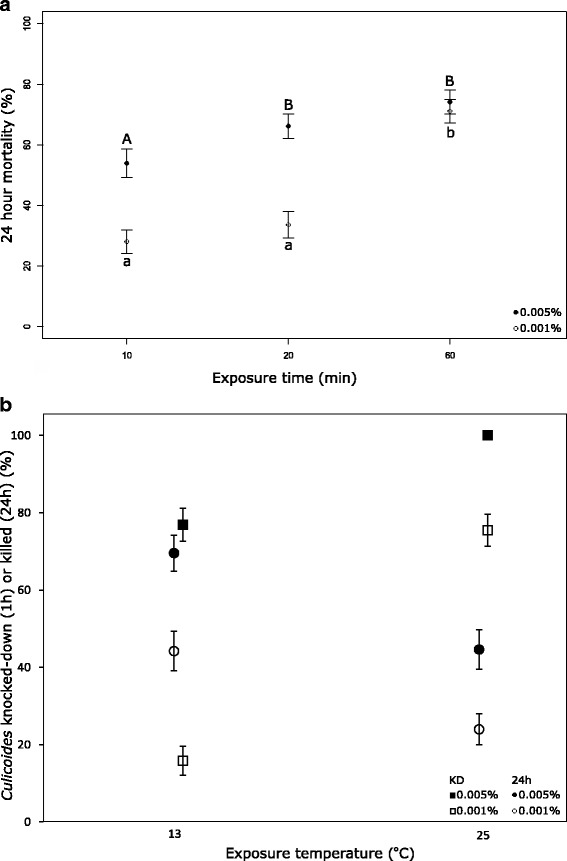
Effects of exposure time and temperature on *C. nubeculosus.* Effect of exposure time on 24-h mortality (**a**) and temperature on knockdown (KD) and 24-h mortality (**b**) on *C. nubeculosus* after exposures to two concentrations of deltamethrin in WHO tubes. Bars indicate standard errors (A, B: *z* = -1.975, *P* < 0.05 (10–20 min), *z* = -3.238, *P* < 0.005 (10–60 min); a, b: *z* = -6.976, *P* < 0.0001 (10–60 min), *z* = -5.785, *P* < 0.0001 (20–60 min) (all *df* = 17))

A significant overall reduction in 24-h mortality was observed when *C. nubeculosus* were exposed to insecticide-treated papers for 10 or 20 min rather than the standard test exposure of 60 min (*z* = -7.225, *z* = -4930, *df* = 35, *P* < 0.0001), but no significant difference was recorded in mortality between 10 min or 20 min exposure. Within each concentration, the 24-h mortality rate at 0.001% deltamethrin was lower after 10 or 20 min exposure compared to 60 min (*z* = -6.976 and *z* = -5.785, *df* = 17, *P* < 0.0001), but at 0.005% deltamethrin, mortality rates between 20 and 60 min exposure did not differ, with 24-h mortality rates were both higher than that recorded in the 10 min exposure (*z* = -1.975, *df* = 17, *P* < 0.05 and *z* = -3.238, *df* = 17, *P* < 0.005 respectively; Fig. 3a).

#### Effect of temperature

A greater knockdown effect was recorded at 25 °C than at 13 °C (*z* = -8.544, *df* = 19, *P* < 0.0001). However, after 24 h following exposure, the mortality recorded was significantly greater at 13 °C than at 25 °C (*z* = 4.384, *df* = 19, *P* < 0.0001; Fig. 3b).

#### Contact irritancy and spatial “repellency”

**Fig. 4:**
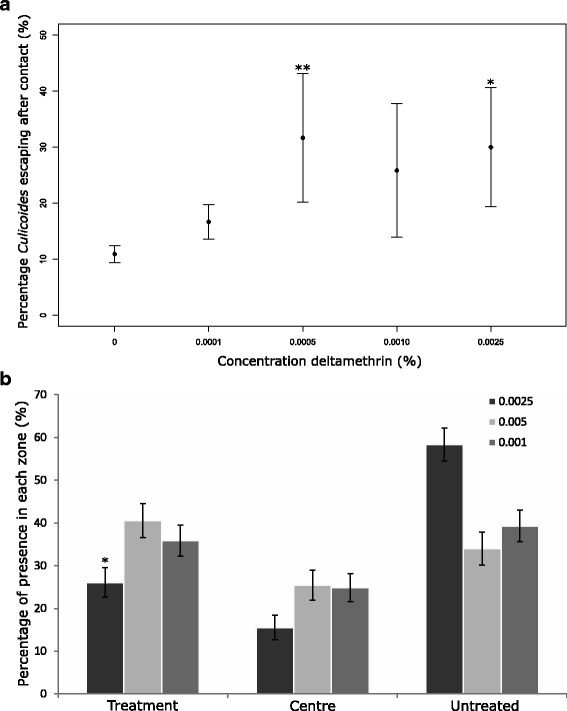
Effects of deltamethrin on contact irritancy and spatial repellency on *C. nubeculosus.* Contact irritancy as measured by the percentage of *C. nubeculosus* escaping deltamethrin treatment after contact (**a**) and spatial repellency recorded as the percentage of *Culicoides* moving from the centre into the zone with the control paper, as explained in Fig. 1 (**b**). Bars indicate standard errors. *P < 0.05; **P < 0.01

There was no significant difference in response according to the orientation of the treatment tube during control experiments, indicating that there was no intrinsic bias in attraction. Significantly more *C. nubeculosus* moved away from the deltamethrin-treated paper after contact at concentrations of 0.0025% (*χ* ^2^ = 13.34, *df* = 5, *P* < 0.05) and 0.005% (*χ* ^2^ = 15.31, *df* = 5, *P* < 0.01; Fig. 4a). Evidence of spatial repellency was only found at 0.0025% deltamethrin (*χ* ^2^ = 4.38, *df* = 1, *P* < 0.05; Fig. 4b).

### Experiments with field-caught *Culicoides* in Tamil Nadu, India

#### Dose-response with deltamethrin

**Fig. 5:**
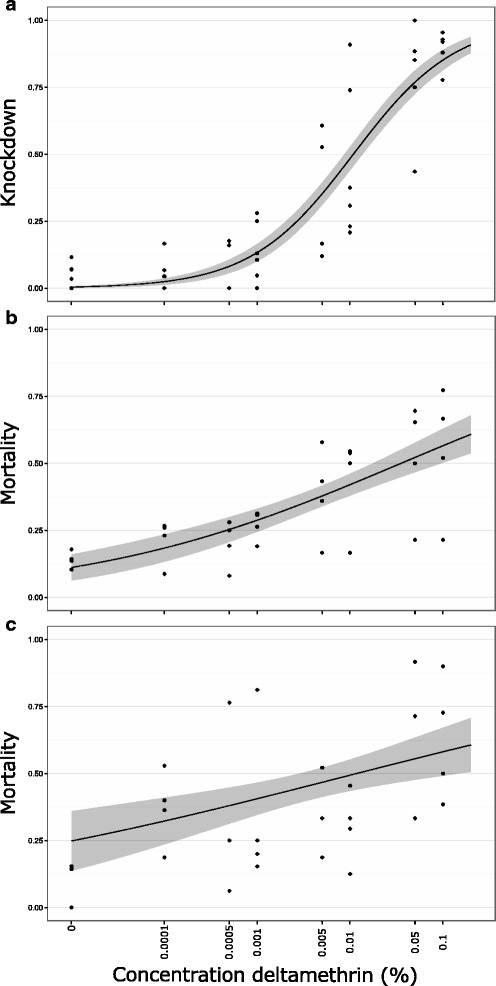
Knockdown and mortality of field caught *Culicoides* in India. One-hour knockdown (**a**) and 6-h mortality (**b**) of Indian *Culicoides* sp. and 24-h mortality of *Culicoides oxystoma* (**c**) after exposure to deltamethrin in WHO tubes. Tested insects were collected in the field using an OVI trap and exposed to different concentrations of deltamethrin. Grey bands indicate 95% confidence intervals

There was a positive correlation between deltamethrin concentration and both knockdown and 6-h mortality (Fig. 5a, b). Knockdown after 1 h resulted in an LC_50_ of 0.011% [0.008, 0.013] (3.96 mg/m^2^) and the LC_90_ was 0.178% [0.100, 0.256] (36 mg/m^2^). Six hours after exposure, some of the *Culicoides* that had been knocked down regained activity, resulting in greater LC values (LC_50_:3.54*10^-2^% [7.04*10^-3^, 6.37*10^-2^] (12.73 mg/m^2^); LC_90_: 2.01*10^2^ % [0, 8.01*10^2^] (72.48 g/m^2^)). The most abundant species in the field-collections was *C. oxystoma* and therefore analyses were repeated for this species only. For *C. oxystoma* the 24 h deltamethrin LC_50_ was 1.17*10^-2^% [0, 2.99*10^-2^] (4.32 mg/m^2^) and LC_90_ was 2.77*10^4^ % [0, 3.23*10^5^] (9.98 kg/m^2^) (Fig. 5c).

#### Repellent effects of deltamethrin, neem oil and neem smoke

There was no significant effect of neem smoke or neem oil on *Culicoides* catches (total catch: *F*_(2,23)_ = 0.27, *P* = 0.769; females only: *F*_(2,23)_ = 0.27, *P* = 0.762; Table 1). There was also no significant difference in trap catches between sheep treated with neem oil, deltamethrin or untreated sheep (total catch: *F*_(2,43)_ = 0.36, *P* = 0.699; females only: *F*_(2,43)_ = 0.45, *P* = 0.642; Table 1).

**Table 1:** Comparative effects of neem oil and neem smoke and deltamethrin and neem oil on the number of *Culicoides* sp. caught in CDC-LED traps (means and 95% CIs) (*n* = 9)

	log(total catch)	log(females)
Neem oil and neem smoke		
Control	4.82 (2.49–7.15)	4.52 (2.25–6.79)
Neem oil	5.24 (2.88–7.60)	4.94 (2.65–7.23)
Neem smoke	5.93 (3.57–8.29)	5.61 (3.32–7.90)
Deltametrin and neem oil		
Control	4.73 (2.87–6.59)	4.29 (2.13–6.45)
Deltamethrin	4.40 (2.54–6.26)	3.93 (1.77–6.09)
Neem oil	4.84 (2.98–6.70)	4.45 (2.30–6.60)

#### Effect of neem oil and deltamethrin-treated fleece on *Culicoides* survival

There was no significant effect of treatment (exposure to either neem oil or deltamethrin) or time on *Culicoides* knockdown or either 6 or 10 h mortality when compared to untreated controls (*P* > 0.05).

## Discussion

### Laboratory studies

Laboratory reared *C. nubeculosus* were highly susceptible to deltamethrin, but the LC_90_ in our study was higher than that recorded for the same species in another study [16]. This may be a result of keeping the tubes in our study in an upright position during exposure as prescribed by the WHO for use with mosquitoes, which may have caused less contact between the *C. nubeculosus* and the insecticide-treated paper. Despite this, the concentrations required to achieve 90% mortality were lower than those that are theoretically achieved during application to livestock in the field (e.g. in the use of pour-on formulations). However, this does not account for uneven spread of the insecticide over the animal’s body (as previously demonstrated by [21]). In this case, the concentration of deltamethrin that *Culicoides* are exposed to on certain parts of the treated host may be too low to prevent biting and onwards transmission of disease [9].

In our study we demonstrated that mortality was significantly reduced and the LC_50_ and LC_90_ values were higher when the exposure time was reduced. In exposure trials on human beings the majority of *Culicoides* feed immediately following introduction to a host [17], so the lower exposure times were aimed to be more representative of the exposure time in the field than the hour long exposure previously used. It means that previous studies may have overestimated the effect of the insecticide. Similarly, we found that temperature significantly affected the effect on knockdown and mortality. *Culicoides* are more readily knocked down when exposed to deltamethrin at higher temperatures, which may be a result of an increased metabolic rate. There was greater mortality at low temperatures, which may be explained by lower activity levels and therefore longer contact with the treated papers. This lower temperature is representative of temperatures at which BTV transmission is likely to cease in the field due to a lack of viral replication, inhibited by temperature-dependent polymerase activity [22]. During outbreaks of BTV in temperate regions, the temperature following import of potentially viraemic ruminants is a key factor in risk assessment. At 13 °C, animals would still be subject to treatment with insecticides following import and hence the reduced effect demonstrated here may influence the probability of onwards transmission of BTV in the field. These findings highlight the importance of creating realistic testing conditions in laboratory studies that can be more accurately translated to the field.

### Field study

In our field study in India, we found low mortality rates and high LC_50_ and LC_90_ values which suggest resistance in *C. oxystoma* populations to deltamethrin although the LC values fell outside the tested range of concentrations and hence had to be extrapolated. Resistance to insecticides has been documented for *Culicoides*, although this has largely been reported in the USA where frequent treatment of larval habitats with organophosphate or organochlorine-based products has occurred (see [6] for review). In India, resistance could occur for the same reason in areas where intensive regimes of insecticide use are maintained against biting insects. *Culicoides oxystoma* is a suspected vector of BTV in India [4], and comparative studies of the other major putative vector species in this region would be useful in placing toxicity in context, particularly as they include *C. imicola* for which baseline data has already been collected from areas lacking sustained insecticide use [23, 24].

### Repellency

The main function of deltamethrin is to be toxic to the target insect upon contact; however it also has repellent properties [25] and there was evidence of both irritancy and “spatial repellence” of deltamethrin on *C. nubeculosus* behaviour in our laboratory study. The actual contact irritancy is expected to be higher because *Culicoides* that are knocked down upon initial contact with the treated papers are not able to move away from the insecticide. However, when tested in the field, deltamethrin did not affect the number of *Culicoides* species collected in trap catches. This could be due to behavioural resistance to the spatial effect of deltamethrin in the field populations of *Culicoides* or due to species-specific differences in behavioural in response to deltamethrin. Other studies with *Culicoides* have also found that repellent effects of insecticides vary with product, concentration and species [25]. Similarly, the traditional methods (neem oil and burning of neem leaves), used by subsistence farmers in India in our study did not result in a significant difference in *Culicoides* trap catches. While providing important preliminary data, a challenge in these studies was controlling for the large number experimental factors that could account for this result; however, including the concentration of active ingredient used, the method of presentation and the spatial arrangement of compost heaps, water taps and neem trees on the site.

## Conclusions

Insecticide application on livestock is often used as a first barrier to reduce BTV transmission [6]. However, transmission of BTV has been shown to occur even when deltamethrin has been used [9, 26]. Although our studies demonstrate that deltamethrin can cause mortality and spatial repellency under laboratory conditions, this does not necessarily translate to the field. The lack of efficacy of deltamethrin, as well as the local traditional methods, in our Indian field studies suggest that local populations may have developed some form of physiological and behavioural resistance to the compound, possibly due to consistent exposure of populations to pesticides. Further field studies are needed develop effective methods of controlling *Culicoides* in India, and to investigate the resistance status of local populations.

## Additional file

Additional file 1: WHO insecticide tests original datafile. (XLSX 26 kb)

## Abbreviations

BTV: Bluetongue virus
LC: Lethal concontration
WHOPES: World Health Organisation Pesticide Evaluation Scheme
